# Distinct roles of the anaphylatoxin receptors C3aR, C5aR1 and C5aR2 in experimental meningococcal infections

**DOI:** 10.1080/21505594.2019.1640035

**Published:** 2019-07-16

**Authors:** Marcel Muenstermann, Lea Strobel, Andreas Klos, Rick A. Wetsel, Trent M. Woodruff, Jörg Köhl, Kay O. Johswich

**Affiliations:** a Institut für Hygiene und Mikrobiologie, Universität Würzburg, Würzburg, Germany; b Institut für Medizinische Mikrobiologie und Krankenhaushygiene, Medizinische Hochschule Hannover, Hannover, Germany; c Institute of Molecular Medicine Center for Immunology and Autoimmune Diseases, The University of Texas Health Science Center, Houston, TX, USA; d School of Biomedical Sciences, The University of Queensland, Brisbane, Australia; e Institute for Systemic Inflammation Research, University of Lübeck, Lübeck, Germany; f Division of Immunobiology, Cincinnati Children’s Hospital and University of Cincinnati College of Medicine, Cincinnati, OH, USA

**Keywords:** C3a, C5a, C3aR, C5aR1, C5aR2, meningococcal disease, sepsis, inflammation

## Abstract

The complement system is pivotal in the defense against invasive disease caused by *Neisseria meningitidis* (*Nme*, meningococcus), particularly via the membrane attack complex. Complement activation liberates the anaphylatoxins C3a and C5a, which activate three distinct G-protein coupled receptors, C3aR, C5aR1 and C5aR2 (anaphylatoxin receptors, ATRs). We recently discovered that C5aR1 exacerbates the course of the disease, revealing a downside of complement in *Nme* sepsis. Here, we compared the roles of all three ATRs during mouse nasal colonization, intraperitoneal infection and human whole blood infection with *Nme*. Deficiency of complement or ATRs did not alter nasal colonization, but significantly affected invasive disease: Compared to WT mice, the disease was aggravated in *C3ar*^−/-^ mice, whereas *C5ar1*^−/-^ and *C5ar2*^−/-^ mice showed increased resistance to meningococcal sepsis. Surprisingly, deletion of either of the ATRs resulted in lower cytokine/chemokine responses, irrespective of the different susceptibilities of the mice. This was similar in *ex vivo* human whole blood infection using ATR inhibitors. Neutrophil responses to *Nme* were reduced in *C5ar1^−/-^* mouse blood. Upon stimulation with C5a plus *Nme*, mouse macrophages displayed reduced phosphorylation of ERK1/2, when C5aR1 or C5aR2 were ablated or inhibited, suggesting that both C5a-receptors prime an initial macrophage response to *Nme*. Finally, *in vivo* blockade of C5aR1 alone (PMX205) or along with C5aR2 (A8^Δ71−73^) resulted in ameliorated disease, whereas neither antagonizing C3aR (SB290157) nor its activation with a “super-agonist” peptide (WWGKKYRASKLGLAR) demonstrated a benefit. Thus, C5aR1 and C5aR2 augment disease pathology and are interesting targets for treatment, whereas C3aR is protective in experimental meningococcal sepsis.

## Introduction

Although usually a harmless colonizer of the upper respiratory tract, the Gram-negative bacterium *Neisseria meningitidis* (*Nme*, meningococcus) is feared for its potential to cause rapidly progressing invasive diseases such as meningitis and septicemia [1]. Initial symptoms of invasive meningococcal diseases (IMD) are often not timely recognized before the disease rapidly progresses, thereby limiting the time window for successful treatment; in fact, most deaths caused by meningococcal septicemia occur within 24 h after hospital admission [2]. The incidence of IMD is highest in infants and toddlers (<5 years), with a fatality rate around 5–20%. Approximately 20% of survivors suffer permanent damage, including neuronal and cognitive defects, deafness, chronic pain and limb amputations [2].

In order to survive and replicate in the bloodstream, *Nme* must avoid killing by the complement system. In fact, *Nme* evolved intricate mechanisms to counteract complement effector functions [3]. The major virulence factor of *Nme* in protecting against complement is a polysaccharide capsule, by which 12 different serogroups can be distinguished [4]. Furthermore, *Nme* sequester the complement regulator fH via the outer membrane proteins fHbp [5] and NspA [6]. Both, capsule and fHbp are used as antigens in meningococcal vaccines [7,8].

The complement system is by far the most important branch of the innate immune system protecting against IMD. Protection critically depends on membrane attack complex (MAC) mediated lysis, as evidenced by the extremely enhanced risk of infection in individuals with defects in terminal complement components required for the MAC assembly [9]. While the role of the MAC in meningococcal disease is well characterized, there is only limited knowledge about the impact of other effector mechanisms triggered by the complement system. Particularly, the roles of the pro-inflammatory mediators C3a and C5a, which are small cleavage fragments liberated during complement activation, have drawn very little attention thus far. These so-called “anaphylatoxins” bind to and thereby activate their corresponding cellular receptors, the C3aR, C5aR1 and C5aR2 (formerly called C5L2) [10]. These “anaphylatoxin receptors” (ATRs) belong to the superfamily of G-protein-coupled receptors, and their activation triggers a multitude of innate immune and inflammatory responses. These include granulocyte and monocyte chemotaxis as well as degranulation, mast cell degranulation [10], vasodilation and endothelial activation [11,12], modulation of cytokine release [13], modulation of T-helper cell polarization [14] and tissue macrophage activation [15].

C5a is the most potent anaphylatoxin, driving a predominantly pro-inflammatory potential response through C5aR1 activation [16]. C5aR1 is a G-protein coupled receptor highly expressed on neutrophil granulocytes and its activation aggravates chronic and acute inflammatory conditions, such as sepsis [17], ischemia-reperfusion injury [18], inflammatory bowel disease [19] and others. In accordance, C5aR1 has been suggested as a key therapeutic target to treat inflammatory disorders [20,21].

In addition to C5aR1, C5a can also bind to C5aR2, which is uncoupled from G-protein signaling, mainly due to a mutation of the crucial DRY motif [22]. In fact, the functional role of C5aR2 is controversially debated as it has been described as a non-signaling scavenger receptor [23], an anti-inflammatory molecule [24,25], a pro-inflammatory receptor-like C5aR1 [26,27], or as a modulator of C5aR1 and C3aR function [28,29]. In addition, there is data suggesting that C5aR2 is involved in metabolic processes including stimulation of triglyceride synthesis [30].

C3aR is activated only by C3a, thereby inducing chemotaxis of eosinophils, mast cells and macrophages [31,32]. Furthermore, C3a, like C5a, increases vascular permeability [33,34] and contributes to airway hyper-responsiveness [35]. C3aR is implicated in the systemic response to LPS challenge, as mice lacking C3aR display enhanced susceptibility and higher levels of IL-1β [36]. Similarly, C3aR deficient mice are more susceptible to infection with the Gram-positive bacterium Listeria monocytogenes [37]. C3aR expression on neutrophils appears to be predominantly anti-inflammatory, by inhibiting neutrophil mobilization into the blood [38]. Thus, although often termed a pro-inflammatory receptor-like C5aR1, C3aR has many anti-inflammatory facets. Therefore, it has recently been suggested to refer to C3aR as an inflammatory modulator [39].

Multiple inflammatory pathways are activated during meningococcal disease [40], which could be modulated by the anaphylatoxins and their receptors. A contribution of the anaphylatoxin receptor family members to the inflammation-driven disease pathology in meningococcal disease appears likely. In support of this, our previous work demonstrated a significant C5aR1-driven aggravation of experimental *N. meningitidis* sepsis in a mouse model as well as in human whole blood [41].

Here, we aimed to extend our analyses to the relative contributions of all three anaphylatoxin receptors, C3aR, C5aR1 and C5aR2 to meningococcal nasopharyngeal colonization as well as sepsis pathology using *in vivo* and *ex vivo* models of disease.

## Results

### Asymptomatic Nme colonization is not significantly affected by complement or ATRS in CEACAM1-humanized mice

**Figure 1.**
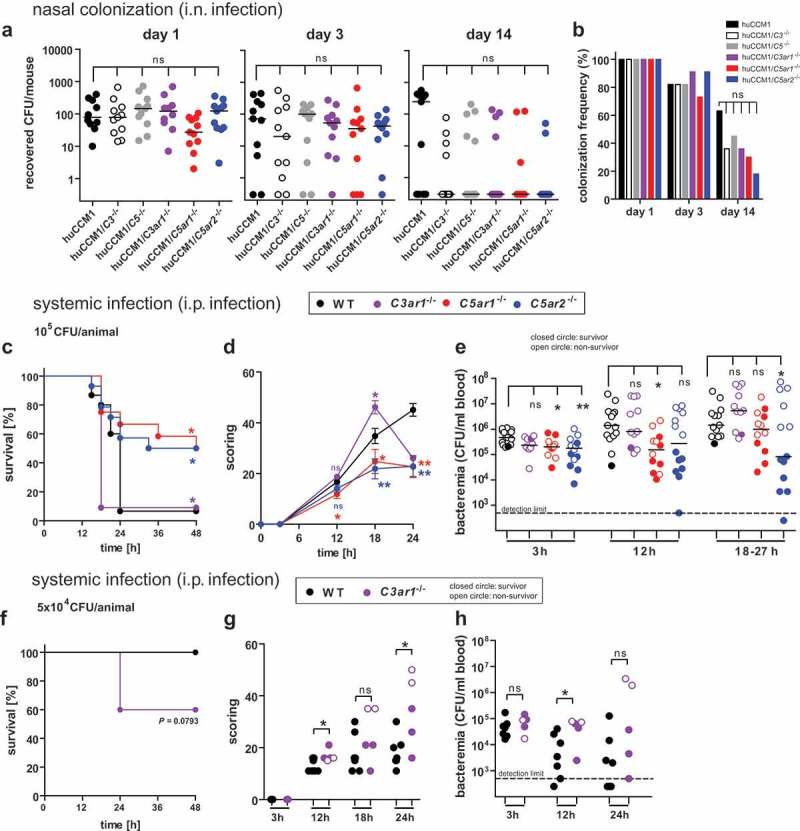
Impact of complement and ATRs on asymptomatic nasal colonization following i.n. infection versus invasive disease following i.p. injection of *Nme* in mice. **Nasal colonization** (a,b): Interbreeds of mice expressing human *CEACAM1* with mice lacking C3 (*C3^−/-^*), C5 (*Hc°^/0^*), C3aR (*C3ar1^−/-^*), C5aR1 (*C5ar1^−/-^*) or C5aR2 (*C5ar2^−/-^*) were intranasally infected with 10^5^ CFU of *Nme* and sacrificed at indicated time points (day 1, 3, 14) to sample nasal tissues for viable *Nme* (n = 11 animals per cohort). (a) Recovered *Nme* expressed as CFU per individual animal at indicated time points after infection. (b) Percentage of mice colonized at each time point. **Invasive disease** (**c-h)**: (c,e) 11 to 15 mice of each genotype were i.p. infected with a total dose of 10^5^ CFU of serogroup B *Nme* strain MC58 per mouse and followed for 48 h before cessation of the experiment. (f-h) Seven WT and 5 *C3ar1^−/-^* mice were subjected to a sub-lethal dose of 5 × 10^4^ CFU per animal. (c,f): Survival rates after infection. (d,g) Clinical scoring over time. Note that only the first 24 h is included in the analysis, since only a single animal in the WT and *C3ar1^−/-^* cohort survived past this time point. (e,h) Bacteremia in tail vein blood samples during the course of infection as indicated by the time points below the x-axis. Bacteremia plotted at ’18–27 h’ includes values at 24 h of all mice surviving longer than 27 h and the values of the non-survivors at their time of death (by sacrificing at the “humane endpoint”) within this time window. Closed circles indicate surviving animals, open circles indicate non-survivors. Limit of detection was 500 CFU/ml.

The complement system is the major contributor to the defense against *Nme* in the blood, both in human disease [3] as well as in the mouse model [41]. Yet, IMD is preceded by *Nme* colonization of the nasopharyngeal mucosa, and it is currently unknown whether mechanisms of serum resistance in *Nme* also benefit their mucosal colonization. Thus, we wanted to test whether complement and the anaphylatoxins as important attractors of phagocytes impact on *Nme* mucosal colonization *in vivo*. We, therefore, used our *CEACAM1*-humanized mouse model [42–44] in combination with deficiencies in C3 (*C3^−/-^*), C5 (*C5^−/-^*), C3aR1 (*C3ar1^**-/-**^*) C5aR1 (*C5ar1^−/-^*) or C5aR2 (*C5ar2^−/-^*) to monitor *Nme* nasal colonization following intranasal infection with 10^5^ CFU. Colonization levels of the mice were similar across all genotypes at day 1 and day 3 (Figure 1(a,b)). At day 14, there was a trend to lower bacterial burden and colonization frequency across all mouse lines carrying any complement deficiency compared to the complement-sufficient control strain, but this was not statistically significant. Of note, none of the mice showed signs of disease or a positive blood culture taken 4 h after i.n. infection. In all, complement or the ATRs apparently do not significantly affect nasopharyngeal colonization in the *CEACAM1*-humanized mouse model.

### Deficiency of C5ar1 or C5ar2 ameliorates the course of experimental meningococcal sepsis in mice, whereas deficiency of C3ar worsens it

Previously, we uncovered a significantly increased resistance of *C5ar1^−/-^* mice over wild-type (WT) mice in a murine sepsis model of *N. meningitidis* [41]. To determine whether the other anaphylatoxin receptors, C3aR and C5aR2, also impact on the outcome of experimental meningococcal sepsis, we compared the course of disease in cohorts of WT, *C3ar1^−/-^, C5ar1^−/-^* and *C5ar2^−/-^* mice. In accordance with our earlier work, *C5ar1^−/-^* mice showed significantly enhanced survival in comparison to WT mice (Figure 1(c)). Interestingly, a similar beneficial effect was observed with *C5ar2^−/-^* mice, whereas, in striking contrast, *C3ar1^−/-^* mice succumbed even faster to the disease. Accordingly, *C5ar1^−/-^* and *C5ar2^−/-^* mice displayed reduced clinical scores than WT mice, whereas *C3ar1^−/-^* showed aggravated symptoms during the course of the disease (Figure 1(d)). Past 18 h, there was only one single survivor in the *C3ar1^−/-^* cohort, which displayed only moderate symptoms. Aggravation of disease was paralleled by the increased bacterial burden in the diseased mice (Figure 1(e)). At 3 h, *C5ar1^−/-^* and *C5ar2^−/-^* showed reduced meningococcemia as compared to WT, and this was also seen at 12 h for *C5ar1^−/-^* and around 24 h for *C5ar2^−/-^*. In contrast, meningococcemia was not significantly different between WT and *C3ar1^−/-^* mice at any time point. In general, higher levels of meningococcemia were found in non-survivors (indicated by open symbols) than in survivors (filled symbols), reflecting that active replication of *N. meningitidis* correlates with disease outcome. In survivors, the bacterial counts decreased further after 24 h.

In order to elaborate the enhanced susceptibility of *C3ar1^−/-^* mice, an infection with a lower inoculum was conducted, demonstrating higher mortality (Figure 1(f)), aggravated symptoms (Figure 1(g)) and enhanced bacterial burden in the blood (Figure 1(h)) among *C3ar1^−/-^* mice compared to WT mice.

Taken together, mice lacking C3aR are more vulnerable to experimental meningococcal sepsis than WT mice, whereas mice lacking either of the two C5a-receptors are more resistant.

### C3ar, C5ar1 and C5ar2 impact similarly on cytokine response during experimental meningococcal sepsis in mice

**Figure 2.**
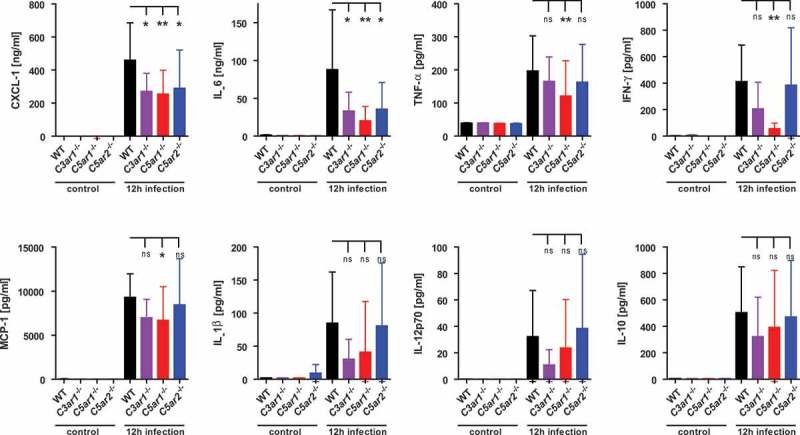
Cytokine and chemokine response of *C3ar1^−/-^, C5ar1^−/-^* and *C5ar2^−/-^* mice 12 h after intraperitoneal infection with *N. meningitidis*. Tail vein blood samples from the same mouse cohorts as in Figure 1(c-e) were analyzed for inflammatory cytokines and chemokines at 12 h after infection. Controls were samples from the same mice one week prior to the experiment. Plotted are means ± standard deviation.

Anaphylatoxin receptors are known to modulate the cytokine response of immune cells [45–47] as well as endothelial cells [13]. Our previous study showed that mice lacking C5aR1 mounted a significantly weaker cytokine response during *N. meningitidis* sepsis than WT mice, indicating a positive modulation of cytokine release by C5aR1 during infection [41]. Here, we analyzed the cytokine profiles in tail vein blood plasma samples taken 12 h post infection from WT, *C3ar1^−/-^, C5ar1^−/-^* and *C5ar2^−/-^* mice (Figure2). Consistent with our earlier work [41], *C5ar1^−/-^* mice displayed significantly reduced levels of CXCL-1, IL-6, TNF-α, IFN-γ, and MCP-1, in comparison to WT mice. *C5ar2^−/-^* mice showed significantly lower levels of CXCL-1 and IL-6 than WT mice, whereas all other tested cytokines and chemokines were similar to WT mice. Given that *C3ar1^−/-^* mice showed the highest susceptibility to experimental sepsis, with aggravated symptoms and high levels of bacteremia (Figure 1), we were surprised that their cytokine/chemokine response was lower than, or similar to, that in WT mice. However, the differences were only significant for CXCL-1 and IL-6, whereas a trend was observed throughout the entire panel of mediators released in response to infection. Interestingly though, the difference in the plasma levels of CXCL-1 between WT and either of the knockout mice was already seen as early as 3 h after infection (supplementary Fig. S1). This indicates that early events in innate immune recognition prime a differential ATR-dependent chemokine/cytokine response in the mice.

These data point towards a similar net contribution of all three anaphylatoxin receptors to the cytokine/chemokine output in the mice, suggesting that the exacerbated inflammatory response *per se* is not strictly determining the course of disease in the mice.

### N. Meningitidis driven neutrophil activation is attenuated in C5ar1^-/-^ mice, but not in C3ar1^-/-^ or C5ar2^-/-^ mice.

**Figure 3.**
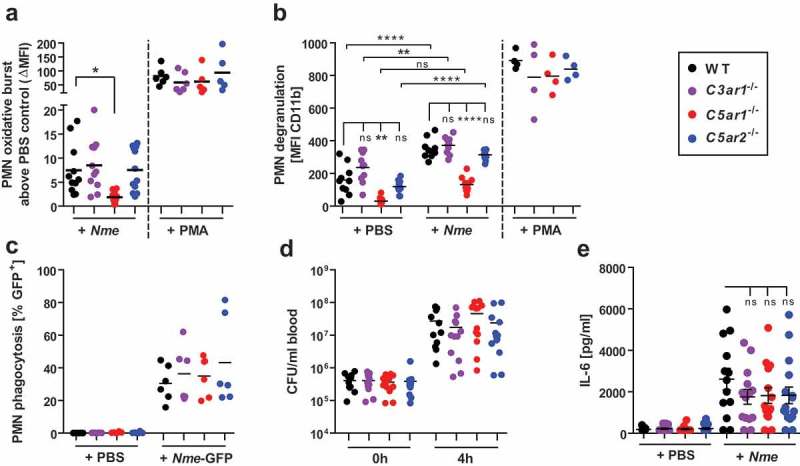
Neutrophil responses of *C3ar1^−/-^, C5ar1^−/-^* and *C5ar2^−/-^* mice in *ex vivo* whole blood infection. Hirudin-anticoagulated whole blood was infected either with *Nme* or stimulated with PMA versus PBS as a negative control. Samples were incubated at 37°C rotating over top. (a) The oxidative burst response of mouse neutrophils as assessed by the DHR123-assay. Blood samples were treated for 1 h either with PBS, 10^7^ CFU/ml of *Nme* or with 100 nM PMA as a positive control. Values of the PBS-condition for each individual mouse sample were subtracted from the others in order to correct for the intrinsic GFP-signal of cells from *C5ar2^−/-^* mice. Thus, the y-axis plots ΔMFI values. (b) Neutrophil degranulation as measured by an increase of CD11b on the cell surface. Blood samples were treated for 1 h with PBS (negative control), 10^7^ CFU/ml *Nme* or with 100 mM PMA (positive control). Values obtained with the isotype-control for the anti-CD11b antibody were subtracted in order to correct for the intrinsic GFP-signal of cells from *C5ar2^−/-^* mice. (c) Phagocytosis of GFP-expressing *Nme* by neutrophils in mouse whole blood. Blood samples were infected with 10^8^ CFU/ml *Nme* and incubated for 1 h and GFP-signal of the cells measured by flow cytometry. In order to correct for the intrinsic GFP-signal of *C5ar2^−/-^* neutrophils, the mean MFI difference between WT and *C5ar2^−/-^* was subtracted from all *C5ar2^−/-^* neutrophils. (d) Survival of *Nme* in mouse blood. Whole blood samples were infected with 10^5^ to 10^6^ *Nme* and samples were taken immediately (0 h) or after 4 h of incubation rotating at 37°C (4 h) and serial dilutions plated on blood agar plates for CFU enumeration. (e) IL-6 plasma levels at 4 h after infection of mouse whole blood with 10^6^ CFU/ml of *Nme* strain MC58.

In searching for mechanisms explaining the disparate outcomes of *Nme* sepsis in the individual ATR-knockout mice, we first focused on the functional responses of neutrophils, *i.e*. the cell type featuring the highest expression of C5aR1 and C5aR2 [10]. In fact, unbridled neutrophil activation is deemed detrimental during sepsis, and this has been particularly attributed to the C5a/C5aR1 axis [17,48]. In order to determine differences in neutrophil responses in the ATR-deficient mouse strains, we used a hirudin-based whole blood model for *ex vivo* infection, as this anticoagulant does not interfere with complement activation or *Nme* growth [49,50]. Upon infection with *Nme*, the neutrophils showed an oxidative burst response (Figure 3(a)), degranulation (Figure 3(b)), and they engulfed GFP-expressing *Nme* (Figure 3(c)). Consistent with our earlier observations [41], neutrophils from *C5ar1^−/-^* mice showed a significant reduction in oxidative burst and degranulation, whereas there were no significant differences between the mouse genotypes when stimulation was done with PMA as a positive control. However, *C3ar1^−/-^* and *C5ar2^−/-^* neutrophils mounted comparable oxidative burst and degranulation responses as those from WT mice. Phagocytosis of *Nme* by neutrophils was similar for all four mouse genotypes, and likewise, bacterial counts rose similarly among all mouse genotypes in blood incubated *ex vivo* for 4 h. In order to ascertain the role of complement in phagocytic uptake of *Nme* by neutrophils in the murine model, we compared *Nme* phagocytosis in WT versus *C3*^−/-^ whole mouse blood. In agreement with our recent reports [41,51], phagocytosis is severely impaired in the absence of complement (supplementary Fig. S2). The fact that *Nme* can actually grow in mouse blood despite ongoing neutrophil responses and phagocytosis demonstrates that neutrophil responses alone are insufficient to clear the infection, despite their significant *in vivo* contribution to *Nme* clearance reported earlier [41]. In accordance with the attenuated cytokine response *in vivo* (Figure 2), IL-6 responses in the *ex vivo* whole blood infection model showed a (non-significant) down-trend for all ATR-knockout mice compared to WT (Figure 3(e)).

Taken together, we found that only *C5ar1^−/-^* mice showed reduced neutrophil responses to *Nme* whole blood infection, whereas *C3ar1^−/-^* and *C5ar2^−/-^* neutrophils responded comparably to those from WT mice.

### C5ar1 and C5ar2 modulate phosphorylation of ERK1/2 in early macrophage activation

**Figure 4.**
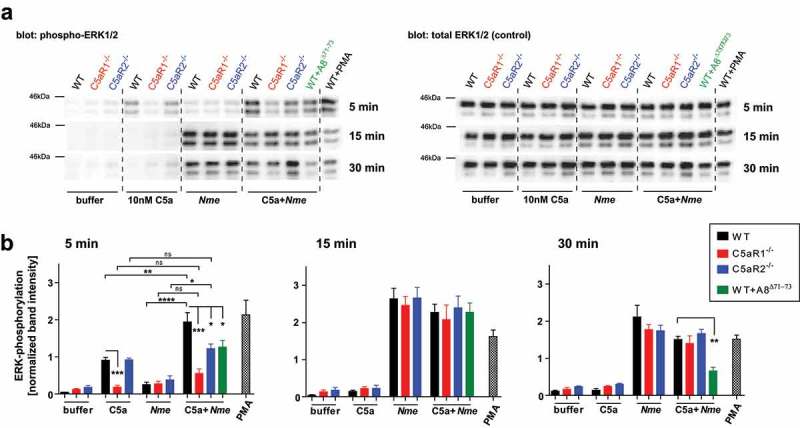
ERK1/2 phosphorylation in bone marrow-derived murine macrophages in response to C5a and/or *Nme*. Macrophages were derived from WT, *C5ar1^−/-^* or *C5ar2^−/-^* mice and differentiated for 8 days in L929-conditioned medium and their phenotype (CD68^+^CD11b^+^F4/80^+^) verified by flow cytometry. Cells were stimulated with murine C5a (10 nM final concentration), *Nme* (heat-inactivated; corresponding to 10^8^/ml), or both, for the indicated duration (5 min, 15 min, 30 min) before harvesting samples. (a) Western blots probed with anti-phospho-ERK1/2 (left panels) or with anti-ERK1/2 (right panels) as a loading control. (b) Densitometric analysis of the Western blot bands from four independent experiments.

C5aR1 and C5aR2 differ fundamentally in their ability to signal via G-proteins to trigger cellular responses (*e.g*. neutrophil chemotaxis) [22], which is reflected by the distinct neutrophil responses in whole blood (Figure 3). In attempting to find a common denominator for the homogenously ameliorated disease phenotype of *C5ar1^−/-^* and *C5ar2^−/-^* mice (Figure 1) as compared to WT mice, we measured ERK1/2 phosphorylation, a central hub in cellular signaling events upon extracellular stimuli. Indeed, C5aR1 and C5aR2 both act on ERK phosphorylation in neutrophils, although for C5aR2, there is conflicting data regarding whether it is activating or inhibiting ERK1/2 phosphorylation [22,28,29]. We challenged bone marrow-derived murine macrophages with 10 nM C5a and/or 10^8^ CFU/ml heat-inactivated *Nme*. Robust ERK1/2 phosphorylation in response to C5a alone occurred in WT and *C5ar2^−/-^* macrophages within 5 min, but not in *C5ar1^−/-^* macrophages (Figure 4). ERK1/2 phosphorylation in response to C5a was of short duration and declined to baseline levels within 15 min. In contrast, there was no significant ERK1/2 phosphorylation within 5 min when the cells were exposed to *Nme* alone. Yet, WT macrophages showed a significant increase of ERK1/2 phosphorylation when C5a along with *Nme* was added, whereas this was not observed with *C5ar1^−/-^* or *C5ar2^−/-^* macrophages. Also, blocking both C5a-receptors with A8^Δ71−73^ did not significantly heighten ERK1/2-phosphorylation above the C5a alone condition. Thus, at this early timepoint, both C5a-receptors appear to function in concert to prime a fast and robust ERK1/2 phosphorylation in macrophages. At later timepoints, there was no difference among the genotypes upon stimulation with *Nme* alone or *Nme* plus C5a. Interestingly, at 15 min and 30 min, there was a slight (not significant) reduction in ERK1/2 phosphorylation when the cells were stimulated with *Nme* plus C5a as compared to *Nme* alone. Also, treatment with A8^Δ71−73^, limited the duration of ERK1/2 phosphorylation, as it was significantly reduced in comparison to the WT control at 30 min.

From these data, we conclude that ERK1/2 phosphorylation in macrophages is positively influenced by C5aR1 and C5aR2 during the very early phase of their activation by C5a and *Nme*. This provides a possible approach to explain the similar outcome of *in vivo* disease despite different abilities of the two C5a-receptors in coupling to G-proteins and activation of neutrophil responses.

### Pharmacologic targeting of C5a-receptors, but not C3aR ameliorates experimental meningococcal sepsis

**Figure 5.**
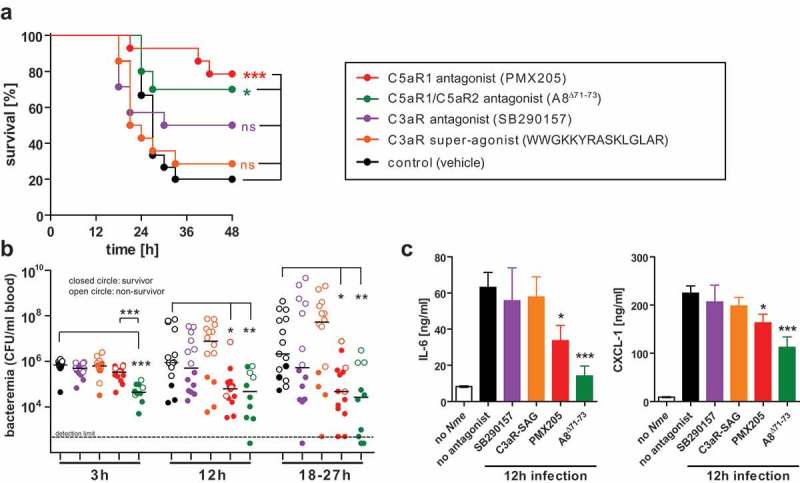
Experimental *Nme* sepsis in WT mice treated with agonists or antagonists of the anaphylatoxin receptors. Mice were subjected to intraperitoneal treatment with antagonists to C5aR1 (PMX205), C5aR1 plus C5aR2 (A8^Δ71−73^), C3aR (SB290157), or with a super-agonist (WWGKKYRASKLGLAR) of C3aR, or with vehicle alone (sterile 5% glucose solution) starting prior to infection and continued throughout the experiment (see Methods). Each mouse was intraperitoneally infected with a total of 10^5^ CFU of *Nme*. Mice were monitored for 48 h before the experiment cessation. (a) Survival curves of mice. (b) Bacteremia at indicated time points of the mice. Bacteremia plotted at ’18–27 h’ includes values at 24 h of all mice surviving longer than 27 h and the values of the non-survivors at their time of death (by sacrificing at the “humane endpoint”) within this time window. Closed circles indicate surviving animals, open circles indicate non-survivors. Limit of detection was 500 CFU/ml. (c) Inflammatory mediators IL-6 and CXCL-1 as measured by ELISA in tail vein blood samples obtained from the same mice as in A at 12 h after infection. Plotted are means ± SEM.

Given the differential susceptibility of *C3ar1^−/-^, C5ar1^−/-^* and *C5ar2^−/-^* mice to experimental meningococcal sepsis (Figure 1), and the successful treatment of mouse meningococcal sepsis with a C5aR1-specific antagonist, PMX-205, in our earlier work [41], we speculated that pharmacologic targeting of C3aR or C5aR2 might as well influence the course of disease in WT mice. To test this hypothesis, we injected mice with the antagonist SB290157 to block C3aR, or with the superagonistic peptide WWGKKYRASKLGLAR to stimulate C3aR [52], or with the antagonist A8^Δ71−73^ to simultaneously block C5aR1 and C5aR2, or with PMX205 to block C5aR1 alone before intraperitoneal challenge with a lethal dose of 10^5^ CFU of *Nme*. The control cohort received vehicle only. Consistent with earlier results, PMX205 treatment resulted in a significantly higher survival rate (Figure 5(a)), reduced levels of bacteremia (Figure 5(b)) and lower levels of inflammatory cytokines (Figure 5(c)). A similar effect was observed upon blockade of C5aR1 together with C5aR2 using A8^Δ71−73^. Notably, the effect on bacteremia and cytokine response was even more pronounced with A8^Δ71−73^ as with PMX205, and this difference was statistically significant for bacteremia at 3 h. Thus, there may be a slight-added benefit in blocking both C5a-receptors simultaneously. In contrast, neither antagonizing (SB290157) nor activation (superagonist) of C3aR seemed to significantly alter the course of disease in the treated mice in comparison to the vehicle control.

Considering the small differences between sepsis outcome after blockade of C5aR1 alone (PMX205) versus C5aR1 plus C5aR2 (A8^Δ71−73^), the additional effect of blocking C5aR2 seems to be minor. Unfortunately, no specific C5aR2 inhibitor is available to individually assess the role of C5aR2 during *Nme* sepsis.

Taken together, we conclude from these data that interference with the C5a/C5a-receptor axes ameliorates disease, whereas our results do not support the notion that C3aR is a promising candidate for pharmacologic targeting during IMD.

### Blockade of C3ar, C5ar1, and C5ar2 reduces IL-8 secretion and neutrophil oxidative burst in human whole blood

**Figure 6.**
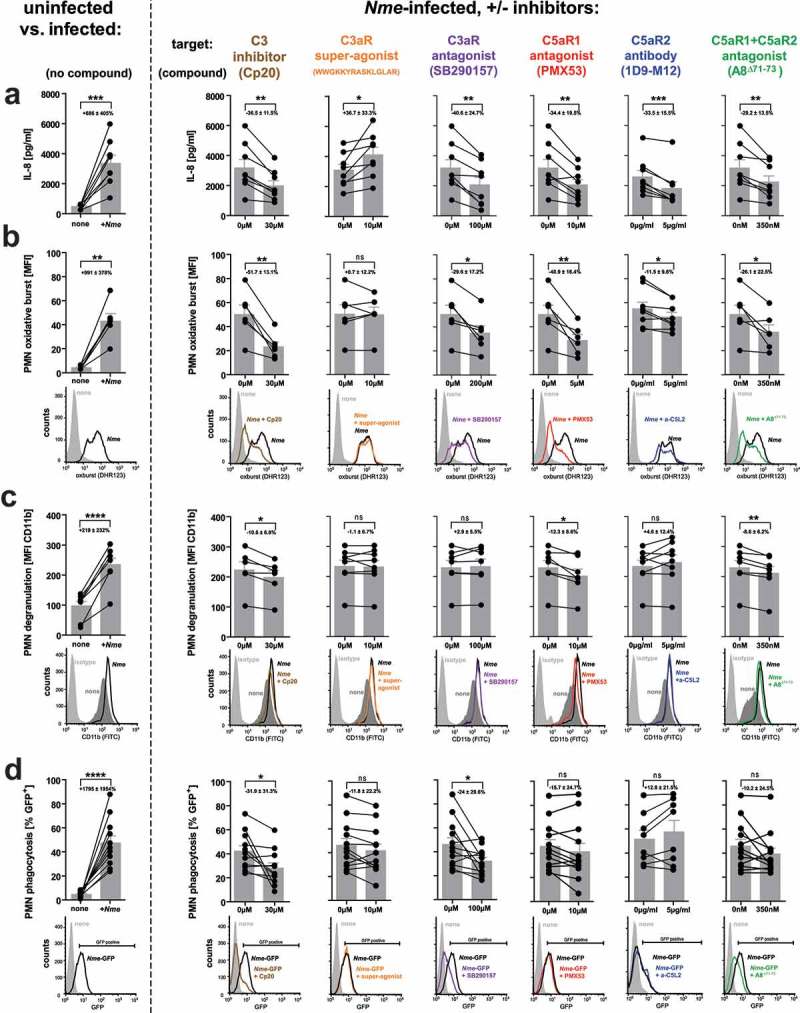
Innate immune responses in human whole blood during infection with *Nme* in presence of specific C3 or ATR antagonists. Hirudin-anticoagulated human blood samples (n = 6 donors) were incubated for 10 min with the indicated antagonists before the addition of *Nme*. The target structures are indicated above the graphs, with the agent indicated in brackets. In the graphs, values obtained from the same individual are represented as dots connected with lines; the bar graphs plot mean ± SEM of the same data. The average reduction of the analyzed response relative to no antagonist addition is given as percentage ± SD in each graph. (a) IL-8 response at 90 min of infection with 10^6^ CFU/ml *Nme* at 37°C rotating over top. (b) Neutrophil oxidative burst as measured by DHR123 assay in human whole blood after infection with 10^7^ CFU/ml *Nme* at 1 h of incubation at 37°C rotating over top. Values are expressed as mean fluorescence intensity (MFI). Representative histograms from the flow cytometric analysis are shown directly below the graphs. (c) Neutrophil degranulation assessed as surface CD11b by flow cytometry. Data are plotted as MFI, representative histograms are shown directly below the graphs. (d) Neutrophil phagocytosis of GFP-expressing *Nme* (10^8^ CFU/ml) as assessed by flow cytometry after incubation in whole blood for 1 h. Date are plotted as % of GFP-positive neutrophils; representative histograms and gating are shown directly below the graphs.

In order to consider the human-specific tropism of *Nme*, we conducted human whole blood infection experiments to determine the influence of the individual anaphylatoxin receptors on cytokine secretion and neutrophil responses. Therefore, we blocked the individual anaphylatoxin receptors in hirudin-anticoagulated blood and measured secretion of IL-8 and neutrophil oxidative burst response upon *ex vivo Nme* infection. The IL-8 secretion in infected human blood (Figure 6(a)) was reduced upon blockade of either C3aR (using SB290157), or C5aR1 (using PMX53), or C5aR2 (using IgG_2a_ mAb clone 1D9-M12), an observation well matching the reduced cytokine/chemokine response of *in vivo* infected knockout mice (Figure 2). In addition, a significant reduction of IL-8 secretion was observed when C5aR1 together with C5aR2 was blocked using A8^Δ71−73^ [53], but the effect did not exceed that of the individual blockade of the individual C5a-receptors. Also, when complement was blocked at the stage of C3 convertase by compstatin Cp20, a significant reduction (36%) was seen, which as well was comparable with the other treatments, which inhibited IL-8 secretion by 29–42%. Compstatin efficiently blocked complement activation, as indicated by the near-complete ablation of C5a liberation (reduction by 87%) in *Nme* infection whole blood from the same donors (supplementary figure S3). Triggering C3aR using the superagonistic peptide WWGKKYRASKLGLAR [52] enhanced IL-8 release in whole blood.

In addition to IL-8 secretion, we found a significant reduction of the neutrophil oxidative burst response (Figure 6(b)) upon inhibition of C3aR, C5aR1, or C5aR2. Simultaneous inhibition of C5aR1 and C5aR2 with A8^Δ71−73^, yielded a significantly decreased burst response. In contrast, the C3aR super-agonist had no effect on the oxidative burst response. When complement activation was blocked using compstatin Cp20, a significant decrease of the neutrophil oxidative burst was observed.

Similar to the impact on oxidative burst, blockade of C3, C5aR1 or simultaneous blockade of C5aR1 and C5aR2 significantly reduced the neutrophil degranulation in whole blood upon *Nme* infection; however, no effect was seen when C3aR was either triggered or inhibited, and C5aR2 inhibition as well did not impact the degranulation response (Figure 6(c)).

Finally, we assessed the uptake of GFP-expressing meningococci by neutrophils in human whole blood. Here, a significant reduction of phagocytosis was only evident upon inhibition of C3 using compstatin Cp20 or blockade of the C3aR, whereas there was no significant effect with any of the other treatments (Figure 6(d)).

Of note, we did not observe a bactericidal effect of any of the used agents to block or activate the ATRs (supplementary Fig. S4).

Taken together, the results obtained in human blood are in good accordance with those of *in vivo* mouse infections and mouse *ex vivo* whole blood infections when considering the effect of all three ATRs in their contribution to cytokine/chemokine release, and, additionally, for C5aR1 with neutrophil activation (oxidative burst, degranulation). However, while we did not observe an effect of C3aR or C5aR2 on mouse neutrophil activation during mouse blood infection, this is apparently the case in human whole blood.

## Discussion

We employed *in vivo* models to analyze the contribution of anaphylatoxin receptors to nasal colonization and invasive disease with *Nme*. While we found no significant contribution of complement in general (C3, C5), or of the ATRs during asymptomatic *Nme* nasal colonization, we identified a significant contribution of the ATRs to the course of *Nme* sepsis in mice. Thus, we expanded upon our recent observation that C5aR1 aggravates *Nme* sepsis pathophysiology [41] in showing that C3aR is protective, whereas C5aR2, like C5aR1, is detrimental in the mouse *Nme* sepsis model. We found that pharmacological blockade of C5aR1 alone, and particularly in combination with C5aR2, significantly ameliorated disease symptoms in mice, whereas tampering with the C3a/C3aR-axis did not benefit the infected mice. Interestingly, invalidation of any of the three ATRs i*n vivo* and/or *ex vivo* similarly reduced the cytokine/chemokine levels in mouse and human blood, suggesting the involvement of pathomechanisms beyond inflammatory mediator release disease progression and outcome. Neutrophil responses in mouse and human whole blood showed a complex and partially species-specific pattern of ATR influences on neutrophil effector functions. When C5aR1 and/or C5aR2 were invalidated in mouse macrophages, early ERK1/2 phosphorylation in response to *Nme* along with C5a was significantly reduced. Hence, we suggest that C5aR1 and C5aR2 triggered ERK1/2 phosphorylation initiates a macrophage response early on during experimental disease, which may underlie the similar disease phenotype found in *C5ar1^−/-^* and *C5ar2^−/-^* mice.

Considering the nasal infection model, earlier work with the *CEACAM1*-humanized mouse model implied a connection between *Nme* nasal colonization exogenous complement activation using Cobra venom factor (CVF) when administered shortly (4 h) but not long (24 h) before intranasal instillation of *Nme* [42]. Thus, we were surprised to see no effect on *Nme* colonization in mice devoid of functional complement (C3 or C5 deficient) here. Thus, we conclude that in the previous work, the injection of CVF may have caused changes to the epithelial environment which transiently (4 h only) affected *Nme* colonization.

Considering the invasive disease model, this study is in agreement with our own previous work [41] showing a deleterious effect of C5aR1 during meningococcal sepsis, since *C5ar1^−/-^* mice showed significantly reduced mortality, morbidity, cytokine/chemokine levels and reduced neutrophil activation patterns in mouse and human blood. Overall, this is consistent with the pro-inflammatory capacity of C5aR1 as the canonical receptor for C5a which is well known to exacerbate sepsis-induced pathomechanisms [48,54].

While the pro-inflammatory potential of C5aR1 is very clear-cut, the biologic effects of its sibling receptors, C3aR and C5aR2, are usually more subtle and their actual contribution to inflammation and immunity is less pronounced [39,55]. In fact, the functional role of C5aR2 has been heavily debated over the past 20 years [55–57]. The detrimental role of C5aR2 during experimental *Nme* sepsis supports the notion of a functionally relevant, pro-inflammatory role for this receptor, as described by Rittirsch *et al*. using the cecum ligation and puncture (CLP) model [26]. The detrimental effects of C5aR1 during sepsis have been linked to neutrophil dysfunction [58]. Yet neutrophil activation can be beneficial (pathogen clearance) as well as deleterious (organ damage) during sepsis [59]. Our data show reduced neutrophil responses in *C5ar1^−/-^* mice, suggesting that C5aR1 might trigger adverse responses in *Nme* sepsis. In contrast, *C5ar2^−/-^* neutrophils responded similar to WT neutrophils in our *ex vivo* analyses, therefore, neutrophils are likely not the cell type that mediates the C5aR2-dependent phenotype in our mouse model. From infections of clodronate liposome-treated mice, we know that macrophages are crucial to control *Nme* sepsis [41]. Given that *C5ar1^−/-^* and *C5ar2^−/-^* macrophages (and WT macrophages treated with A8^Δ71−73^) show decreased ERK1/2 phosphorylation at early timepoints of C5a/*Nme* stimulation, we speculate that the beneficial outcome of experimental *Nme* sepsis in *C5ar1^−/-^* and *C5ar2^−/-^* mice (or A8^Δ71−73^ treated WT) is partially due to this early macrophage activation event. Thus, future work will need to dissect the impact of the C5a-receptors on early macrophage activation during *Nme* sepsis.

In contrast to *C5ar1^−/-^* and *C5ar2^−/-^* mice, *C3aR^−/-^* mice were more susceptible to *Nme* sepsis. Similar observations were made in other infection models, such as LPS challenge [36], *Listeria monocytogenes* infection [60] and *Chlamydia psittaci* infection [61]. Yet, this disease aggravation is not a general feature, since *C3ar1^−/-^* mice are more resistant to *Pseudomonas aeruginosa* pneumonia [60], indicating distinct mechanisms involved in the C3aR-dependent response towards different pathogens. Pharmacological targeting the C3a/C3aR-axis, either by activating (“superagonist”) or by antagonizing (SB293157) C3aR, did not significantly alter the course of the disease as expected based on the results with the *C3ar1^−/-^* mice. Yet, there are some caveats to be considered. If C3aR activation was already at maximum during *Nme* sepsis, the “superagonist” could not increase it any further to alter the disease outcome. Further, the “superagonist” is a linear peptide [52], thereby subject to proteolytic inactivation by serum carboxypeptidase N; hence, its biologic activity could be limited below a therapeutic threshold *in vivo*. On the other hand, the C3aR-antagonist SB290157 has reported off-target activities *in vivo*, causing neutropenia independent to C3aR, as well as having C3aR agonist activity in certain cell settings [62,63]. Thus, we consider our data from *C3ar1-/-* mice most reliable.

Unexpectedly, *C3ar1*^−/-^ mice mounted lower chemokine/cytokine responses as WT mice (CXCL-1 and IL-6) despite aggravated disease symptoms. This finding contrasts previous reports for *C3ar1^−/-^* mice, in which their susceptibility was correlated with inflammatory mediators [36,37,60]. Thus, the functional interrelationship between C3aR and the chemokine/cytokine system differs significantly between infection with *Nme* and other bacterial pathogens. In addition, this suggests that the poor outcome of *C3ar1*^−/-^ mice is not a direct consequence of the aggravated systemic inflammatory response syndrome.

Although mouse neutrophils have been suggested to express C3aR [28,38], recent evidence shows that mouse neutrophils – in contrast to human neutrophils [64] – do not express C3aR [65]. This would explain the lack of a C3aR-dependent phenotype in the mouse whole blood model. Hence, we can only speculate which cell types mediate the beneficial C3aR-dependent effects during *Nme* sepsis. Macrophages of mice and humans express C3aR [47,65] and could be the driving force of C3aR-dependent disease outcome, potentially in a fashion opposing that triggered by the C5a-receptors. Endothelial cells are activated during septic settings and respond to C3a [66] and are crucial interfaces for the interaction between *Nme* and the host [67]. Thus, by modulation of vascular permeability [60] or intravascular coagulation in combination with C3aR-bearing platelets [68], C3aR might impact on the course of *Nme* sepsis in a cytokine/chemokine-independent fashion.

In order to consider the species-restriction of *Nme*, we performed human whole blood infection experiments. While many aspects were well correlated between human and mouse whole blood, few others were in disalignment: The differential effects of C3aR-inhibition on the oxidative burst response and phagocytosis by mouse versus human neutrophils are explained by the lack of C3aR expression in mouse neutrophils [65]. The difference in the oxidative burst response in dependence of C5aR2 may reflect a functional difference in C5aR2 on neutrophils among mice and humans.

*Nme* phagocytosis by neutrophils was similarly unaffected by both C5a-receptors in mice and men, whereas only in human cells, C3aR-blockade had a negative impact. In contrast to other reports on the phagocytosis of *Nme* by human neutrophils [69,70], we did not observe an impact of C5aR1 in this or in our previous study [41]. This may be due to the assay system used (*e.g*. heat-inactivated *Nme* [70] vs. viable *Nme*) or the immune status of the donors (vaccinated [69] vs. unknown immune status). However, we recently reported slightly reduced *Nme* phagocytosis upon C5aR1-inhibition using purified human neutrophils in the presence of immune serum [51], indicating that the whole blood assay may be, although biologically the most relevant, not the most sensitive assay to measure *Nme* phagocytosis. Overall, the importance of neutrophil phagocytosis during *Nme* sepsis is not clear, and future work will have to address this.

In summary, our work demonstrates that the course of experimental *Nme* sepsis is significantly influenced by the anaphylatoxins and their receptors, expanding our knowledge about complement in *Nme* disease. While C3aR activation is protective during experimental *Nme* sepsis, triggering C5aR1 or C5aR2 is harmful. Thus, our data suggest that targeting the C5a/C5aR1-C5aR2 axis could prove useful as an adjunctive treatment in addition to antibiotics to treat human IMD. Also, our data support emerging studies [38,71,72] indicating that the C3a/C3aR-axis acts protectively in many disease contexts.

## Materials and methods

### Animals

Holding of animals was done in accordance with all legal requirements under the German animal welfare act (TierSchVersV). Animal handling and experimentation were approved by the appropriate authorities (government of Lower Franconia) under files 55.2.2531.01–90/13 and 55.2–2532-2–165. All efforts were made to keep animal distress and suffering to a minimum. Animals were housed with a 12 h bright/dark cycle at 22–24°C and 55 ± 10% relative humidity. Testing for specified pathogens as recommended by the Federation for European Laboratory Animal Science Associations (FELASA) was conducted quarterly to ascertain SPF-status of the mice. The mice had *ad libitum* access to water and standard rodent diet and enrichment was placed in all cages. All mice used were on C57Bl/6J genetic background. WT mice were either bred in-house or purchased from Charles-River or Envigo (formerly Harlan). The mouse strains lacking the individual ATRs, namely B6.129S4-*C3ar1*^tm1RWe^ (termed *C3ar1^−/-^*) [36], B6.129S4-*C5ar1*^tm1Cge^ (termed *C5ar1^−/-^*) [73] and B6.129S4-*C5ar2*^tm1Cge^ (termed *C5ar2^−/-^*) [24], and the mouse strain lacking C3 (B6.129S4-C3^tm1Crr^/J, termed *C3^−/-^*) [74], were bred in-house. The mouse strain lacking C5 (B6.FVB-Hc°; here termed *C5^−/-^*) was obtained by crossing the defective gene for C5 (*Hc°*) from the FVB mouse line for 10 generations onto the C57Bl/6J background, as previously described [41]. The transgenic mouse line expressing human CEACAM1 (termed *huCCM1*) [75] was backcrossed onto C57Bl/6J background for 10 generations and was bred in-house.

## Bacteria

*Nme* were cultivated in all assays described below, by plating on Columbia agar containing 5% sheep blood (further referred to as “blood agar”; bioMérieux) and incubated in water-saturated conditions at 37°C with 5% CO_2_. For our experiments, the *Nme* strain MC58, serogroup B (typed B:15:P1.7,16–2:F1-5: ST-74, ST-32 clonal complex) or its capsule-deficient mutant expressing a green fluorescent protein, MC58Δ*csb*-GFP strain [76] was used.

## *Nme* inocula preparation

Meningococci were grown on blood agar plates overnight and transferred onto fresh plates for 4 h to obtain log-phase grown bacteria. The bacteria were then harvested with a cotton swab, resuspended in brain heart infusion broth (BHI). Using a spectrophotometer, OD_600_ of the bacterial suspension was adjusted to 1.0, corresponding to 1.5 × 10^9^ CFU/ml. Further dilutions were made to adjust the concentration to the desired density, as indicated in the specific protocols. For accuracy confirmation, serial dilutions of the inocula were prepared, plated and grown colonies enumerated.

### Nme intranasal infection of mice

The *CEACAM1*-humanized mouse model for *Nme* colonization was described previously [42,44]. In brief, the *Nme* inoculum (strain MC58) was prepared as described above, but in PBS with 1 mM MgCl_2_ instead of BHI, and adjusted to 10^7^ CFU/ml. 10 μl hereof were instillated by dropwise application to both nares of the alert mouse. Mice were monitored for bacteremia at 4 h after infection by spreading 5 µl of tail vein blood onto blood agar plates. Mice were scored using the following scheme to assess clinical scores, which included body weight loss (score “1”: >3% to 5%; score “5”: ≥5% to 10%; score “10”: ≥10% to 20%; score “20”: ≥20%), general physical appearance (score “1”: small aberrations such as slight lack of grooming; score “5”: dull fur, slightly hunched posture; score “10”: dirty fur, hunched posture, smudgy perianal region, dull eyes; score “20”: tremors or shivering, immobility, difficulty to rise from lateral position), behavior (score “1”: minor aberrations; score “5”: impaired motoric mobility; score “10”: self-isolation, lethargy, strongly impaired coordinative abilities; score “20”: unconsciousness, no/minimal defensive reaction, when picked up) and clinical condition (score “1”: small aberrations; score “10”: ±30% changes in breathing frequency; score “20”: ±50% changes in breathing frequency, blood flow impairments). At indicated time points, mice were sacrificed by CO_2_ asphyxiation. Through a tracheal incision, 200 µl PBS with 1 mM MgCl_2_ were applied to collect a nasal wash sample for plating and CFU enumeration. Then, the nose tip was cut off and the nasal mucosa exposed by lateral cuts through the premaxilla. Nasal mucosa was probed by inserting an ultra-fine polyester-tipped aluminum applicator swab (Puritan) wetted with PBS with 1 mM MgCl_2_ and thorough rubbing. The adherent nasal tissues were resuspended in 500 µl of PBS with 1 mM MgCl_2_ to yield a “nasal swab” sample. Nasal wash and nasal swab samples were spread onto *Neisseria-*selective modified Thayer-Martin agar plates and incubated overnight at 37°C, 5% CO_2_ and water-saturated atmosphere before the enumeration of resultant *Nme* colonies.

### Nme induced sepsis in mice

The 6–8-week old male mice were injected intraperitoneally (i.p.) with 200 µl BHI containing 10^5^ CFU/ml (or 5 × 10^4^ CFU/ml, as indicated) of *Nme* and 200 µl iron dextran (Santa Cruz) diluted 1:3.33 in sterile isotonic saline. A second i.p. injection of iron dextran was given 12 h post infection. Mice were weighed and monitored every 3 h for 48 h following the scoring scheme described above. In accordance with the German Animal Welfare Law, mice were humanely sacrificed by CO_2_ inhalation upon reaching the human endpoint criteria (tremors/shivering or inability to rise from lateral position) and were not left to die. To assess plasma cytokine levels, 10 µl tail vein blood was drawn 12 h post infection, mixed with 90 µl PBS containing 5 mM EDTA, centrifuged and the plasma was then frozen at −80°C until ELISA. For bacterial burden assessment, 5 µl tail vein blood was drawn 3 h and 12 h post infection and then every 12 h. The blood was diluted in 45 µl PBS containing 5 U/ml heparin to avoid clotting. Serial 1:10 dilutions were made and plated on Columbia sheep blood agar plates to enumerate colonies after overnight incubation. Additional samples were taken after mouse euthanasia upon reaching the human endpoint.

### Nme sepsis experiments using antagonists and agonists

Mice were infected and monitored as described above. For the blockade of the C5ar1, WT mice received i.p. 3 mg PMX205 per kg of body weight in a total volume of 100 µl per injection at the following time points relative to the infection start: −12 h, −6 h, 0 h, and then every 12 h post infection. In order to block C3aR, WT mice received i.p. 1 mg/kg body weight SB290157 in a total volume of 100 µl per injection every 12 h starting at −12 h. In order to simultaneously block C5aR1 and C5aR2, WT mice received 200 µl of A8^Δ71−73^ at 88,7 µM at −6 h, 0 h, 6 h, 12 h and then every 12 h. In order to stimulate C3aR, WT mice received 1 mg per kg body weight of the C3aR-superagonist peptide (WWGKKYRASKLGLAR) in a total volume of 100 µl per injection at −3 h, 0 h, 6 h, 12 h and then every 12 h. Control WT mice were treated with vehicle alone at −12 h, −6, 0 h, 6 h and then every 12 h. All agonist/antagonist injections were freshly prepared in sterile 5% glucose solution (vehicle) and sterile filtered through 0.2 µm syringe-mounted devices. Sterility of injected compounds was monitored by plating onto Columbia sheep blood agar plates and incubation for 48 h with none of the preparations ever yielding bacterial contamination.

### Mouse whole blood assays to assess oxidative burst in neutrophils:

Mice were euthanized by CO_2_ inhalation and whole blood was drawn via cardiac puncture using hirudin monovettes (525 antithrombin units/ml; Sarstedt). Hirudin was chosen as anticoagulant since it does not interfere with complement or with *Nme* viability [50]. Mouse whole blood samples were infected with 10^7^ CFU/ml of *Nme*; 100 nM PMA (Sigma Aldrich/Merck) served as a positive control, whereas PBS was used as a negative control. Samples were incubated, rotating for 15 min at 37°C. Then, 20 µg/ml dihydrorhodamine 123 (DHR123) (Sigma Aldrich/Merck) were added and the samples were incubated rotating for 1 h at 37°C. Then, samples were placed on ice and stained for 30 min with anti-mouse Ly6G-APC (Clone: 1A8; BioLegend). After hypotonic lysis of the erythrocytes, samples were washed with cold PBS and fixated with 4% formaldehyde for 30 min at RT in the dark. Flow cytometric analysis was done on a BD FACSCalibur (Becton Dickinson) and data analyzed and plotted with FlowJo v10 (FlowJo, LLC). Cell clusters were gated out with an FSC/SSC gate. Neutrophils were gated as Ly6G-APC^high^ and fluorescence intensity of oxidized DHR123 assessed in the FL1-H channel. For further analysis, the PBS background signal in FL1-H was subtracted from the corresponding signals in the PMA and the *Nme* samples to correct for the slight GFP-dependent shift of cells isolated from *C5ar2^−/-^* mice, which express low levels of GFP.

### Mouse whole blood assays to assess degranulation in neutrophils

To measure neutrophil degranulation, mouse whole blood was obtained and infected/stimulated as described for the oxidative burst assay. The samples were incubated for 60 min at 37°C, then placed on ice and then stained either with anti-mouse Ly6G-APC (clone 1A8; BioLegend) plus anti-mouse/human CD11b-FITC (clone M1/70; BioLegend) or with anti-mouse Ly6G-APC (Clone: 1A8) plus FITC labeled rat IgG2b, κ Isotype Ctrl Antibody (clone RTK4530; BioLegend) Neutrophils were gated as Ly6G-APC^high^ and assessed for their expression of CD11b in the FL1-H channel. The values were corrected by subtracting the isotype-FITC background signal of the PBS-controls in FL1-H from the CD11b-signal in all corresponding samples; this subtraction was necessary to correct for GFP-expression in *C5ar2^−/-^* neutrophils.

### Mouse whole blood assay to assess phagocytosis capacity in neutrophils

For the phagocytosis assay, cardiac blood was obtained as described in the oxidative burst assay. The samples were infected with 10^8^ CFU/ml of GFP-expressing *Nme*; to the negative controls, RPMI was added. After incubation for 60 min rotating at 37°C, samples were stained for neutrophils with anti-Ly6G-APC antibody (clone 1A8, BioLegend) for 30 min on ice followed by hypotonic lysis of erythrocytes and fixation of the sample with 4% formaldehyde. Phagocytosis was assessed by flow cytometry on a FACSCalibur (Becton Dickinson) as an increase in FL1-H relative to PBS control. The background subtraction to correct for GFP-expression in *C5ar2^−/-^* neutrophils was done by subtracting the difference between the FL1-H background signal between WT and *C5ar2^−/-^* neutrophils, from the signals in our PBS and *Nme* samples of *C5ar2^−/-^* neutrophils.

### Mouse whole blood assays to assess Nme survival:

Whole blood samples were collected as described for the oxidative burst above and infected with 10^6^ CFU/ml of *Nme*, or treated with PBS as a negative control. Amount of bacteria present in the samples was assessed by dilution plating and colony enumeration at 0 h and 4 h of infection.

### Cytokine analysis using ELISA or bead-based LEGENDPlex^tm^ assay

Plasma samples from infected mice or untreated animals (controls) were either measured for IL-6 and CXCL-1 using DuoSet® ELISA kits (R&D Systems) or using the LEGENDPlex^TM^ mouse inflammation panel immunoassay (BioLegend,) as per the manufacturer’s instructions. Human plasma samples were measured for IL-8 or C5a using DuoSet® ELISA kits (R&D Systems) as per the manufacturer’s instructions.

### ERK1/2-phosphorylation in mouse bone marrow-derived macrophages

Mice were euthanized by CO_2_ asphyxiation and femurs were obtained, placed for 1 min in 70% ethanol and then the opened bones flushed with 15 ml PBS. Cell aggregates were passed through a 25 G needle to obtain single cells and the suspension was passed through a 40 µm cell strainer to remove residual cell clumps and bone fragments. Cells were pelleted at 1000 g for 5 min and then subjected to hypotonic lysis of erythrocytes before washing with 30 ml PBS and centrifugation. Cells were taken up in macrophage differentiation serum (DMEM with 10% fetal calf serum (all Gibco) plus 10% of L929 conditioned medium) [77] and seeded at 10^6^ cells per well in 48 well plates (Corning). Medium was exchanged at days 3 and 6 and macrophage differentiation ascertained as CD68^+^CD11b^+^F4/80^+^ at day 8 by flow cytometry. For analysis of ERK1/2 phosphorylation, cells were used on day 8, when they were confluent. Cells were PBS washed and reaction buffer added (KBR-buffer: 10 mM HEPES, 125 mM NaCl, 1 mM MgCl2, 1 mM CaCl2, pH 7.3 (Virion\Serion) plus 0.1% BSA). After incubation for 30 min for the cells to adjust, the final concentration of 10 nM mouse C5a (R&D Systems), 10^8^/ml heat-inactivated *Nme*, or both, were added. After incubation for 5 min, 15 min or 30 min, the supernatant was removed and cells lysed directly with 1x Laemmli buffer (BioRad) and samples boiled for 10 min. Samples were analyzed on 10% SDS polyacrylamide gels, transferred onto nitrocellulose membranes and probed with rabbit-anti-ERK1/2 (Cell Signaling #9102) or with rabbit-anti-phospho-ERK1/2 (Cell Signaling #9101), followed by goat-anti-rabbit-HRP conjugate (Jackson ImmunoResearch) and enhanced chemoluminescence imaged on a ChemiDoc MP imaging system (BioRad). Analysis of band intensities was done using ImageLab software (BioRad). For data analysis, band intensities were normalized by the division of the phospho-ERK band intensity by the corresponding relative band intensity of the total-ERK blot.

### Human whole blood models

Human whole blood was drawn from healthy donors by venipuncture using hirudin Monovettes (Sarstedt) and used immediately. For measurement of oxidative burst, samples were pre-incubated for 10 min with 20 µg/ml DHR123 (Sigma Aldrich/Merck) and treated by addition of substances as follows: 1 µM PMA (Sigma Aldrich/Merck) as positive control, or 1 µg/ml of C3aR-superagonistic peptide (WWGKKYRASKLGLAR); termed C3aR-SAG here to stimulate C3aR [52], 200 µM SB29015 to inhibit C3aR, 5 µM PMX53 (Tocris Bioscience) to block C5aR1, 50 µg/ml C5aR2-antibody (clone 1D9-M12; mouse IgG2a; sodium azide was removed by repeated dilution of the antibody in PBS followed by concentration using Amicon Ultra centrifugal devices (3 kDa cutoff) (Merck Millipore) to block C5aR2, 350 nM A8^Δ71−73^ to simultaneously block C5aR1 and C5aR2, 30 µM compstatin Cp20 to block complement at the level of C3 convertase [78], or plain RPMI medium (Life Technologies) as negative control. Blood samples were infected with 10^7^ CFU/ml of *Nme* in a final volume of 100 µl and incubated rotating at 37°C for 1 h. After hypotonic lysis of erythrocytes, samples were washed with cold PBS and fixated with 4% formaldehyde for 30 min at RT in the dark and then analyzed by flow cytometry on a FACSCalibur. Cell clusters were gated out with an FSC/SSC gate. Polymorphonuclear cells (PMNs) were gated based on their distinct FSC/SSC profile and oxidative burst assessed as changes in the MFI in the FL1-H channel.

For measurement of IL-8, human whole blood samples treated with antagonists as above were infected with 10^6^ CFU/ml *Nme* and incubated for 90 min before addition of 1 volume ice-cold PBS with 40 mM EDTA to 1 volume of blood, centrifugation at 10,000 g for 5 min at 4°C before harvesting the supernatant. Samples were frozen at −80°C until ELISA analysis.

For measurement of any bactericidal effect of any of the antagonists above, blood samples equipped with concentrations of the antagonists as stated in Figure 3(c) were infected with *Nme* and incubated for 30 min before serial dilutions were made in PBS and plated onto Columbia blood agar plates for incubation and subsequent colony enumeration.

### Ethics statement

For human whole blood experiments, healthy adult donors without any history of meningococcal disease, acute or chronic inflammatory disorders, or antibiotic treatment in the previous 2 weeks were used. The study was conducted in compliance with the Helsinki Declaration. The study protocol was approved by the Ethics Committee of the Medical Faculty of the University of Würzburg (file 181/16-ge) and was carried in accordance with all relevant local guidelines and was in compliance with the Helsinki Declaration. All donors expressed their informed consent in written form.

### Statistics

Statistical analyses of the data were performed using Prism version 6 (GraphPad). For survival analyses (Figures 1(a) and 5(a)), the Mantel–Cox test was used. Non-parametric data (clinical scores) were analyzed using the Kruskal–Wallis test for multiple groups applying Dunnett’s *post hoc* test. Parametric data from more than two cohorts were analyzed by one-way ANOVA applying either Dunnett’s *post hoc* test to compare genotypes or treatments to the corresponding controls (*e.g*. WT in Figures 1(c), 2, 5(b)) or applying Bonferroni’s *post hoc* test to compare all cohorts (Figure 3). For the analysis of bacteremia data (Figures 1(c,f)), 5(b), data were log-transformed to achieve normal distribution of the values. For comparison of two cohorts (Figure 4), the paired, two-tailed Student’s T-test was used. For the analysis of band intensities in Figure 6, one-way ANOVA applying Bonferroni’s *post hoc* test was chosen. Differences were considered to be significant at *P* < 0.05. In all figures, ns denotes not significant, * denotes *P* < 0.05, ** denotes *P* < 0.01, *** denotes *P* < 0.001, **** denotes *P* < 0.0001.

## Data Availability

All data and materials associated with this publication are available upon request.
