# Supplementary material for: Distinct roles of the anaphylatoxin receptors C3aR, C5aR1 and C5aR2 in experimental meningococcal infections
Source: Virulence. 2019 Jul 16;10(1):677–94. doi: 10.1080/21505594.2019.1640035 (PMC6650196; doi:10.1080/21505594.2019.1640035)
Supplement: Supplemental Material [file kvir-10-01-1640035-s001.pdf]

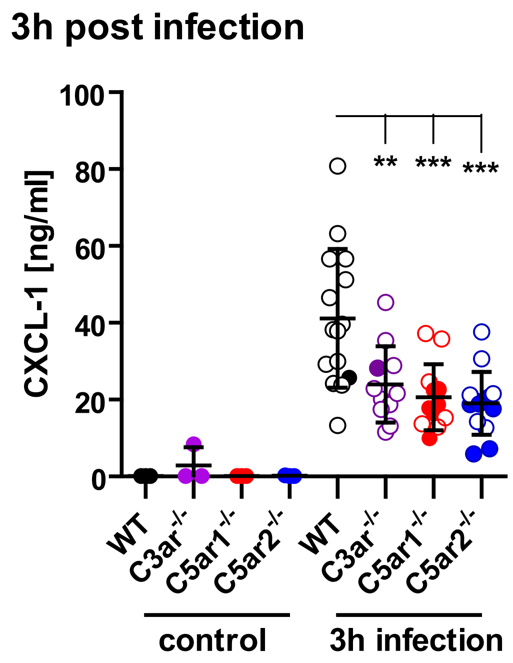

Supplementary figure S1: CXCL-1 plasma levels in WT vs. *C3ar*$^{-/-}$, *C5ar1*$^{-/-}$ and *C5ar2*$^{-/-}$ mice at 3h after intraperitoneal infection with *N. meningitidis* (same cohorts as in Fig.1 and Fig. 2). Each circle represents data from one individual mouse; open circles represent non-survivors, closed circles represent survivors. Lines indicate mean and standard deviation. ** and *** denote $P < 0.01$ or 0.005, respectively, in one-way ANOVA applying Dunnett's multiple *post hoc* test with WT as comparator.

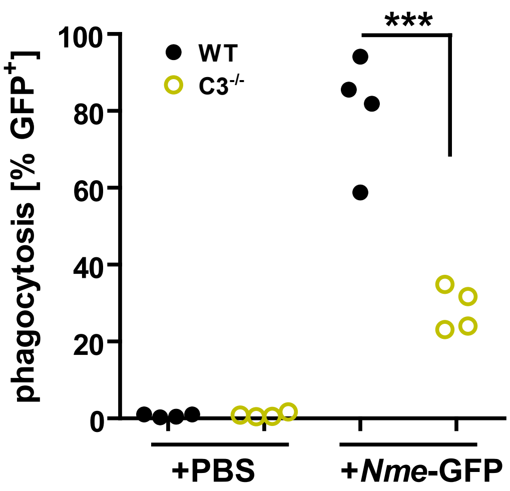

Figure S2: Impact of complement on phagocytosis of *Nme* by neutrophils in whole mouse blood. Hirudin anticoagulated whole blood from WT and C3-deficient mice was incubated for 1h with 10^8 CFU/ml of GFP-expressing meningococci and GFP fluorescence of neutrophils (gated as Ly6G-high) was analyzed by flow cytometry. Plotted is the percentage of cells above PBS-control. Note the dramatic decline in phagocytosis in absence of functional complement in mouse blood of C3-deficient mice.

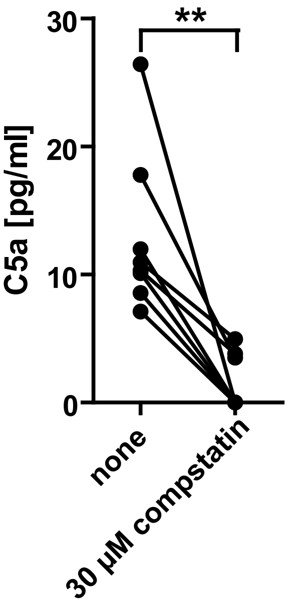

Figure S3: Efficient blockade of *Nme*-induced complement activation by compstatin Cp20.
Hirudin anticoagulated whole blood from human donors was incubated with 10^6 CFU/ml of meningococci with or without addition of the complement C3 inhibitor compstatin Cp20 (30µm final concentratino). After 30 min, 20 mM EDTA was added, and plasma levels of C5a generated upon complement activation under infection conditions measured by ELISA.

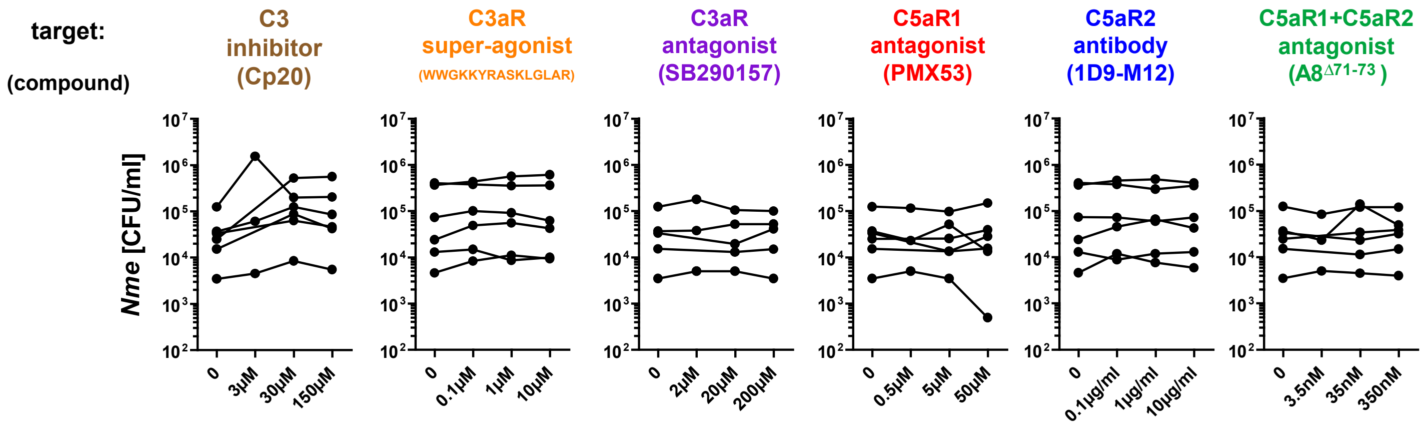

Figure S4: No effect of complement- or ATR-antagonists or C3aR super-agonist on *Nme* viability in human blood. Hirudin anticoagulated whole blood from donors was incubated with 10^6 CFU/ml of meningococci with different concentrations of the compounds (as indicated in graphs). After 30 min, samples were serially diluted, spread onto Columbia sheep blood agar plates and CFU enumerated.
